# Impact of Advanced Radiotherapy on Second Primary Cancer Risk in Prostate Cancer Survivors: A Nationwide Cohort Study

**DOI:** 10.3389/fonc.2021.771956

**Published:** 2021-11-26

**Authors:** Marie-Christina Jahreiß, Wilma D. Heemsbergen, Bo van Santvoort, Mischa Hoogeman, Maarten Dirkx, Floris J. Pos, Tomas Janssen, Andre Dekker, Ben Vanneste, Andre Minken, Carel Hoekstra, Robert J. Smeenk, Inge M. van Oort, Chris H. Bangma, Luca Incrocci, Katja K. H. Aben

**Affiliations:** ^1^ Department of Radiotherapy, Erasmus MC Cancer Institute, Rotterdam, Netherlands; ^2^ Department of Research, Netherlands Comprehensive Cancer Organization, Utrecht, Netherlands; ^3^ The Netherlands Cancer Institute, Radiation Oncology, Amsterdam, Netherlands; ^4^ Department of Radiation Oncology (Maastro), GROW Institute for Oncology and Developmental Biology, Maastricht, Netherlands; ^5^ Department of Radiation Oncology, Radiotherapiegroep, Deventer, Netherlands; ^6^ Department of Radiation Oncology, Radboud University Medical Center, Nijmegen, Netherlands; ^7^ Department of Urology, Radboud University Medical Center, Nijmegen, Netherlands; ^8^ Department of Urology, Erasmus University Medical Center, Rotterdam, Netherlands; ^9^ Research Institute for Health Sciences, Radboud University Medical Center, Nijmegen, Netherlands

**Keywords:** prostate cancer, second primary cancer, survivorship, advanced external beam radiotherapy, three-dimensional conformal radiotherapy

## Abstract

**Purpose:**

External Beam Radiotherapy (EBRT) techniques dramatically changed over the years. This may have affected the risk of radiation-induced second primary cancers (SPC), due to increased irradiated low dose volumes and scatter radiation. We investigated whether patterns of SPC after EBRT have changed over the years in prostate cancer (PCa) survivors.

**Materials and Methods:**

PCa survivors diagnosed between 1990-2014 were selected from the Netherlands Cancer Registry. Patients treated with EBRT were divided in three time periods, representing 2-dimensional Radiotherapy (RT), 3-dimensional conformal RT (3D-CRT), and the advanced RT (AdvRT) era. Standardized incidence ratios (SIR) and absolute excess risks (AER) were calculated to estimate relative and excess absolute SPC risks. Sub-hazard ratios (sHRs) were calculated to compare SPC rates between the EBRT and prostatectomy cohort. SPCs were categorized by subsite and anatomic region.

**Results:**

PCa survivors who received EBRT had an increased risk of developing a solid SPC (SIR=1.08; 1.05-1.11), especially in patients aged <70 years (SIR=1.13; 1.09-1.16). Pelvic SPC risks were increased (SIR=1.28; 1.23-1.34), with no obvious differences between the three EBRT eras. Non-pelvic SPC were only significantly increased in the AdvRT era (SIR=1.08; 1.02-1.14), in particular for the 1-5 year follow-up period. Comparing the EBRT cohort to the prostatectomy cohort, again an increased pelvic SPC risk was found for all EBRT periods (sHRs= 1.61, 1.47-1.76). Increased non-pelvic SPC risks were present for all RT eras and highest for the AdvRT period (sHRs=1.17, 1.06-1.29).

**Conclusion:**

SPC risk in patients with EBRT is increased and remained throughout the different EBRT eras. The risk of developing a SPC outside the pelvic area changed unfavorably in the AdvRT era. Prolonged follow-up is needed to confirm this observation. Whether this is associated with increased irradiated low-dose volumes and scatter, or other changes in clinical EBRT practice, is the subject of further research.

## Introduction

Prostate Cancer (PCa) is the second most commonly diagnosed cancer in men. The worldwide PCa burden is expected to grow to almost 2.3 million new cases by 2040 (1). Considering the overall success in detecting, diagnosing, and treating PCa, the assessment of long-term adverse events of the available treatment options has become increasingly important. A rare but severe long-term adverse event is a radiation-induced second primary cancer (SPC) (2, 3). The associations between radiation exposure and SPC are well-recognized (4, 5). Large cohort studies exploring SPC risk after PCa have confirmed that RT is associated with increased SPC risk (2, 5–10). The majority of these large cohort studies are based on data from national cancer registries in which details on treatment, such as type of External Beam Radiotherapy (EBRT), are typically not registered.

A large proportion of PCa patients receive EBRT. EBRT has undergone major changes over the past decades. In the early 1990s, 2-dimensional radiotherapy (RT) with rectangular fields including the pelvic area was the conventional technique applied. By the second half of the 1990s, there was a shift to 3-dimensional conformal RT (3D-CRT), targeting only the prostate +/- the seminal vesicles. In the Netherlands, from 2005 onwards, intensity modulated RT (IMRT) gradually replaced 3D-CRT. This was closely followed by the introduction of volumetric modulated arc therapy (VMAT). With these advanced techniques, more conformal dose distributions with steeper dose gradients can be achieved. This is done by using multiple intensity-modulated beams, allowing better sparing of the organs at risk, and dose-escalation to the tumor without exceeding critical dose levels to nearby organs (11, 12). IMRT and VMAT are nowadays often combined with daily image-guidance to track the tumor position. These advanced radiotherapy (AdvRT) techniques result in a larger body volume being exposed to low levels of radiation. Studies and theoretical reports have expressed concerns that this may be associated with increased long-term risks of developing a radiation-induced SPC (11, 13, 14).

Clear evidence from clinical observations on the impact of AdvRT on SPC risk is lacking. Few studies exist that explore SPC risk after EBRT, and those studies show inconclusive results (11, 15–17). The aim of the current study is to assess in a large nationwide cohort the risks and time trends of developing SPC after EBRT compared to reference populations, by studying different time periods related to major landmarks in EBRT developments.

## Methods

### Data and Patient Selection

For this retrospective cohort study, data of PCa patients were retrieved through the Netherlands Cancer Registry (NCR). The NCR, established in 1989 with nationwide coverage, is a registry containing data of all new cancer diagnoses in the Netherlands. Notifications of newly diagnosed malignancies are primarily obtained from the nationwide network and registry of histology and cytopathology (PALGA). Information on malignancies without any histological confirmation are extracted from Dutch Hospital Data (DHD). Additional relevant data (patient/tumor characteristics and treatment) are routinely extracted from the hospital patient files. Cancers are coded according to The International Classification of Diseases for Oncology (ICD-O-3) (18). Patients diagnosed between 1990-2014 with a PCa (ICD-O-3 Topography code C61) were included in this study. Information on patient characteristics, as well as information on the primary PCa such as date of diagnosis, morphology, disease stage (Tumor Lymph Node Metastasis (TNM) classification), and treatment, were obtained from the NCR. PCa treatment was classified as follows: EBRT +/- hormonal therapy (HT), radical prostatectomy, brachytherapy, systemic therapy (HT or chemotherapy), active surveillance, and other.

### Definition of Time Periods

Time periods were defined and used as a proxy for the different RT modalities applied. In the early 1990s, 2D-RT was the golden standard and was only gradually replaced by 3D-CRT towards the end of the decade. Therefore, the first time period was defined from 1990 to 1996. The second time period, in which 3D-CRT was the main RT modality, was defined from 1998-2005. In 2005, IMRT was introduced in the Netherlands, which was closely followed by the introduction of VMAT in 2008. The last time period was thus defined from 2008-2014. The introduction of a new RT technique is a gradual process. Hence, to avoid excessive overlap in applied RT modality, some years were disregarded.

### Definition of SPC and Follow-Up Time

All invasive SPC (except for non-melanoma skin cancers) and non-invasive bladder cancer, were included. Information regarding the topography, morphology and date of diagnosis were obtained from the NCR. Analyses were carried out for all SPC, all solid SPC, all hematological SPC and SPC within different anatomical regions (e.g., pelvic and non-pelvic region) and for specific tumor subsites. In general, only the first SPC cancer was included in the analyses. However, for all analyses focusing on a specific group (i.e., solid cancers, hematological cancers, anatomical region of specific subsite), the first SPC cancer within that group was included in the analyses. Hence, the total number of SPC in the overall group does not add up to the sum of SPCs by subsites. Follow-up time was defined as the time between PCa diagnosis until the date of SPC diagnosis, date of death, date of emigration or end of study (31.12.2019), whichever occurred first. SPC diagnosed simultaneously with PCa or within one year after the initial PCa diagnosis were excluded, as these are likely to represent synchronous cancers.

### Statistical Analysis

A descriptive overview including all PCa patients was provided, followed by an overview of the risk of developing a SPC. Standardized Incidence Ratios (SIRs) were calculated to evaluate the risk of SPC in the PCa patient cohort compared to the Dutch population. This was done by dividing the observed number of SPC by the expected number of cases (based on the sex, age, and calendar specific incidence rates in the Netherlands). Poisson regression was used to compute 95% confidence intervals (CI). To measure the excess burden of SPC, absolute excess risks (AER) were calculated. The AER represents the additional incidence beyond the background incidence found in the Dutch general population. It is defined as the difference between the observed and the expected number of patients with a SPC, divided by the number of person years (py) at risk, multiplied by 10,000.

Subsequent analyses focused on the sub cohort of patients with localized PCa (T1-T3N0/X, M0/X) treated with EBRT +/- HT. This cohort was limited to patients with localized disease, as patients with a more advanced stage of disease are likely to experience relapse. We also excluded patients being diagnosed with a T4 or N+ or M+ tumor, in order to minimize the likelihood that the radiation field included the pelvic lymphatic system. Consequently, they are more likely to receive additional treatment, which could not be accounted for, as this information is not available in the NCR. SIRs were calculated by (previously defined) time period, age group (≤70 or >70 years) and for follow-up years for the different time periods. Stratification by time period was done to investigate whether SPC patterns have changed over time, i.e. over the three defined RT periods. Analysis was adjusted for age and calendar year of diagnosis.

Finally, we assessed the relative risk of developing a SPC after RT treatment by comparing the EBRT +/- HT cohort to patients treated with radical prostatectomy. The Fine and Gray method for estimating relative risk (sub-Hazard ratios (sHRs)) was used (19). The relative risk was also estimated per age group and time period of diagnosis. The model was adjusted for age and year of diagnosis. The cumulative incidence of developing a SPC was estimated with death as a competing risk. This analysis was carried out using STATA version 14 (STATA Corp., Texas, USA). SIR and AER analyses was carried out using SAS version 9.2 (SAS Institute Inc., Cary, NC, USA).

### Role of the Funding Source

The Dutch Cancer Society (project grant 12009), which had no further say in the design, analyses or description of the results provided financial support for this study.

## Results

In this study, all patients diagnosed with PCa between 1990-2014 were included (N=161,003). The median age at diagnosis of PCa was 70.0 years (Interquartile range (IQR):64-75). In **Table 1**, a description of the cohort is presented, overall and by initial treatment. EBRT was the most frequently applied initial treatment (26.1%). Within the EBRT cohort, 93.3% had T1-T3N0/X, M0/X PCa. In the complete cohort, a total of 22,538 SPC were observed until the end of 2019. The median time between PCa diagnosis and the development of a SPC was 5.81 years. Overall, a non-significant decreased risk of developing a solid SPC after PCa diagnosis was found, when compared to the Dutch male general population (SIR (95%CI) = 0.98 (0.97-1.00), AER= -3.19 per 10,000 py) (**Table 2**). However, for pelvic SPC a significant increased risk was observed (SIR=1.08 (1.05-1.11); AER=3.40). This was mainly attributed to a significant increase in SPCs in the bladder (SIR=1.08 (1.04-1.11); AER=1.95) and rectum (SIR=1.10 (1.05-1.15); AER=1.17). For hematological SPC, an increased risk was found (SIR 1.09 (1.05-1.14, AER=1.78). In **Supplementary Table 1** additional SPC information for various tumor sites is displayed.

**Table 1:** Patient and tumor characteristics for the complete cohort and per treatment modality.

	Complete Cohort		EBRT +/- HT		Radical Prostatectomy		Brachytherapy		Active Surveillance		Systemic treatment*		Other Treatment	
	n	%	n	%	n	%	n	%	n	%	n	%	n	%
Total	161003	100	42069	26.13	27784	17.26	8036	4.99	26083	16.20	39280	24.40	17851	11.09
Median Age (IQR)	70 (64-75)		70.0 (65-75)		64 (59-67)		65 (61-70)		72 (65-77)		74 (67-79)		73 (67-79)	
Age Group														
<60	19275	11.96	3832	9.11	7442	26.79	1565	19.47	2377	9.11	2830	7.20	1229	6.88
60-69	61191	37.98	16908	40.19	16680	60.03	4304	53.56	8440	32.36	9766	24.86	5093	28.53
70-79	61514	38.18	19999	47.54	3611	13.00	2129	26.49	11081	42.48	16951	43.15	7743	43.38
80+	19123	11.87	1330	3.16	51	0.18	38	0.47	4185	16.04	9733	24.78	3786	21.21
Time Period														
1991-1997	27635	19.75	7176	19.71	2403	9.89	101	1.55	2491	11.06	10058	29.23	5406	34.29
1998-2005	49695	35.51	14946	41.04	8016	32.99	2277	34.83	5976	26.54	13657	39.69	4823	30.59
2006-2014	62616	44.74	14295	39.25	13880	57.12	4159	63.62	14050	62.40	10695	31.08	5537	35.12
Second Primary Cancer (SPC)														
	22538	100	7654	33.96	4131	18.33	1338	5.94	3543	15.72	3435	15.24	2437	10.81
Disease Stage														
T1-2 N0/X, M0/X	111456	69.18	27071	64.35	26187	94.25	7940	98.81	24140	92.55	11882	30.25	14236	79.75
T3 N0/X, M0/X	20002	12.42	12172	28.93	1312	4.72	85	1.06	1155	4.43	4293	10.93	985	5.52
T4 or N+ or M+	29645	18.40	2826	6.72	285	1.03	11	0.14	788	302	23105	58.82	2630	14.73
Median time between PCa diagnosis and SPC (years)	5.81		6.04		7.36		6.65		5.27		4.16		5.54	

External beam radiotherapy with/without hormonal therapy (EBRT +/- HT), *Systemic treatment mainly concerns hormonal therapy only.

**Table 2:** SIRs and AER (per 10,000 person years) for PCa patients treated with EBRT +/- hormonal therapy for different time periods and age groups.

	All Ages				Age ≤70	Age >70
	Obs	Exp	SIR (95%CI)	AER	SIR (95%CI)	SIR (95%CI)
All Solid	6540	6062.9	1.08 (1.05-1.11)*	14.56	1.13 (1.09-1.16)*	1.03 (0.99-1.07)
1991-1996	1128	1069.3	1.05 (0.99-1.12)	10.19	1.10 (1.02-1.19)*	0.99 (0.91-1.09)
1998-2005	2872	2649.1	1.08 (1.04-1.12)*	15.81	1.11 (1.05-1.16)*	1.06 (1.00-1.12)
2008-2014	1591	1452.6	1.10 (1.04-1.15)*	17.23	1.15 (1.07-1.24)*	1.05 (0.98-1.13)
All hematological	889	729.8	1.22 (1.14-1.30)*	4.39	1.23 (1.12-1.35)*	1.19 (1.08-1.31)*
1991-1996	134	104.9	1.28 (1.07-1.51)*	4.80	1.37 (1.09-1.70)*	1.17 (0.88-1.53)
1998-2005	385	311.1	1.24 (1.12-1.37)*	4.97	1.25 (1.09-1.43)*	1.22 (1.04-1.42)*
2008-2014	206	172.8	1.19 (1.03-1.37)*	3.98	1.22 (0.98-1.51)	1.17 (0.97-1.40)
Non-Pelvis	4841	4657	1.04 (1.01-1.07)*	5.50	1.08 (1.04-1.12)*	0.99 (0.95-1.03)
1991-1996	822	817.4	1.01 (0.94-1.08)	0.78	1.04 (0.95-1.13)	0.97 (0.87-1.07)
1998-2005	2098	2037.5	1.03 (0.99-1.07)	4.18	1.05 (1.00-1.12)	1.00 (0.93-1.07)
2008-2014	1206	1115.9	1.08 (1.02-1.14)*	11.06	1.14 (1.05-1.24)*	1.03 (0.96-1.12)
Pelvis	2004	1559.8	1.28 (1.23-1.34)*	13.12	1.37 (1.30-1.46)*	1.22 (1.15-1.30)*
1991-1996	357	278.4	1.28 (1.15-1.42)*	13.21	1.43 (1.25-1.64)*	1.11 0.93-1.30)
1998-2005	929	690.1	1.35 (1.26-1.44)*	16.37	1.39 (1.27-1.51)*	1.30 (1.17-1.43)*
2008-2014	440	362.5	1.21 (1.10-1.33)*	9.37	1.29 (1.11-1.48)*	1.16 (1.02-1.32)*
Bladder	1393	1046	1.33 (1.26-1.40)*	10.18	1.43 (1.33-1.53)*	1.27 (1.18-1.37)*
1991-1996	240	189.5	1.27 (1.11-1.44)*	8.43	1.46 (1.24-1.72)*	1.04 (0.84-1.27)
1998-2005	662	465.5	1.42 (1.32-1.53)*	13.37	1.48 (1.33-1.64)*	1.36 (1.21-1.52)*
2008-2014	299	238	1.26 (1.12-1.41)*	7.34	1.36 (1.13-1.62)*	1.19 (1.02-1.38)*
Rectum	569	461.6	1.23 (1.13-1.34)*	3.12	1.32 (1.18-1.46)*	1.15 (1.02-1.31)*
1991-1996	112	80.7	1.39 (1.14-1.67)*	5.17	1.44 (1.11-1.84)*	1.32 (0.97-1.74)
1998-2005	246	203.4	1.21 (1.06-1.37)*	2.86	1.24 (1.05-1.46)*	1.17 (0.95-1.42)
2008-2014	135	109.3	1.24 (1.04-1.46)*	3.07	1.22 (0.94-1.57)	1.24 (0.97-1.56)

*indicates significant SIRs; observed, (Obs); expected, (Exp); standarized incidence ratio, (SIR); absolute excess risk, (AER).

### Comparison of the EBRT Cohort to the General Population

PCa patients with localized PCa treated with EBRT had an estimated SIR for all solid SPC of 1.08 (1.05-1.11), corresponding with an AER of almost 15 additional men diagnosed with a SPC per 10,000 py (**Table 3**). Specifically, the risk for bladder SPC (SIR=1.33 (1.26-1.40), AER=10.18) and rectum SPC (SIR=1.23 (1.13-1.34), AER=3.12) were increased. With regard to the different time periods, the risk for solid SPC in the EBRT cohort increased over the years. For the time period 2008-2014 a SIR of 1.10 (1.04-1.15) was found, whereas the SIR for the time period 1991-1996 was 1.05 (0.99-1.12). A significant increased risk of developing a SPC in the non-pelvic area was only observed for the most recent time period; SIR=1.08 (1.02-1.14). The risk for pelvic and bladder SPC were significantly elevated throughout all time periods, with the highest risks observed in the second time period (SIR=1.35, 1.26-1.44) and (SIR=1.42, 1.32-1.53) for pelvic and bladder SPC respectively. The risk for rectum SPC was significantly elevated for all time periods but appeared highest in the first time period; SIR=1.39 (1.14-1.67) *versus* (SIR=1.21; 1.06-1.37) and (SIR=1.24; 1.04-1.46) for the later time periods.

**Table 3:** Estimated subHazard ratios by gray and fine method (with adjustment for age and year of diagnosis) for the EBRT cohort *versus* the reference cohort prostatectomy.

	EBRT +/- HT(n)	Radical Prostatectomy(n)	sHRs (95%CI)	p-value
All Solid	6834	3644	1.24 (1.19-1.30)	<0.01*
1991-1996	1172	513	1.25 (1.12-1.40)	<0.01*
1998-2005	2941	1421	1.27 (1.18-1.36)	<0.01*
2008-2014	1735	1174	1.24 (1.14-1.35)	<0.01*
All hematological	949	610	1.03 (0.91-1.15)	0.672
1991-1996	145	74	1.09 (0.80-1.48)	0.605
1998-2005	407	256	0.94 (0.79-1.12)	0.481
2008-2014	254	189	1.09 (0.88-1.35)	0.436
Non-Pelvis	4834	2823	1.14 (1.08-1.20)	<0.01*
1991-1996	814	390	1.13 (0.99-1.29)	0.075
1998-2005	2034	1099	1.13 (1.04-1.23)	<0.01*
2008-2014	1271	913	1.17 (1.06-1.29)	<0.01*
Pelvis	2000	822	1.61 (1.47-1.76)	<0.01*
1991-1996	358	123	1.17 (1.34-2.10)	<0.01*
1998-2005	907	322	1.74 (1.52-2.00)	<0.01*
2008-2014	464	261	1.47 (1.24-1.74)	<0.01*
Bladder	1380	490	1.83 (1.63-2.05)	<0.01*
1991-1996	237	79	1.76 (1.33-2.31)	<0.01*
1998-2005	649	195	2.04 (1.71-2.44)	<0.01*
2008-2014	307	150	1.65 (1.33-2.05)	<0.01*
Rectum	550	312	1.20 (1.03-1.40)	0.023*
1991-1996	110	41	1.51 (1.01-2.27)	0.043*
1998-2005	225	122	1.14 (0.90-1.45)	0.281
2008-2014	145	104	1.16 (0.87-1.55)	0.323

*indicates statistically significant p-values; external beam radiotherapy with/without hormonal therapy (EBRT +/- HT); sub-hazard ratios (sHRs).

Numbers reflect the observed numbers of survivors experiencing the SPC event of interest.

The risk for hematological SPC remained significantly elevated over the different time periods, although it moderately decreased as EBRT advanced [SIR=1.28 (1.07-1.51) to SIR=1.19 (1.03-1.37)]. In **Supplementary Table 2**, SIRs for all subsites and different time periods are displayed.

The age-group specific analysis demonstrated that age is an important factor affecting the risk of SPC. No significant increase of solid SPC was observed for older patients (>70 years), whereas younger patients (≤70) demonstrated a significant increased risk, for solid SPC and other subsites (**Table 2**).

**Figure 1:**
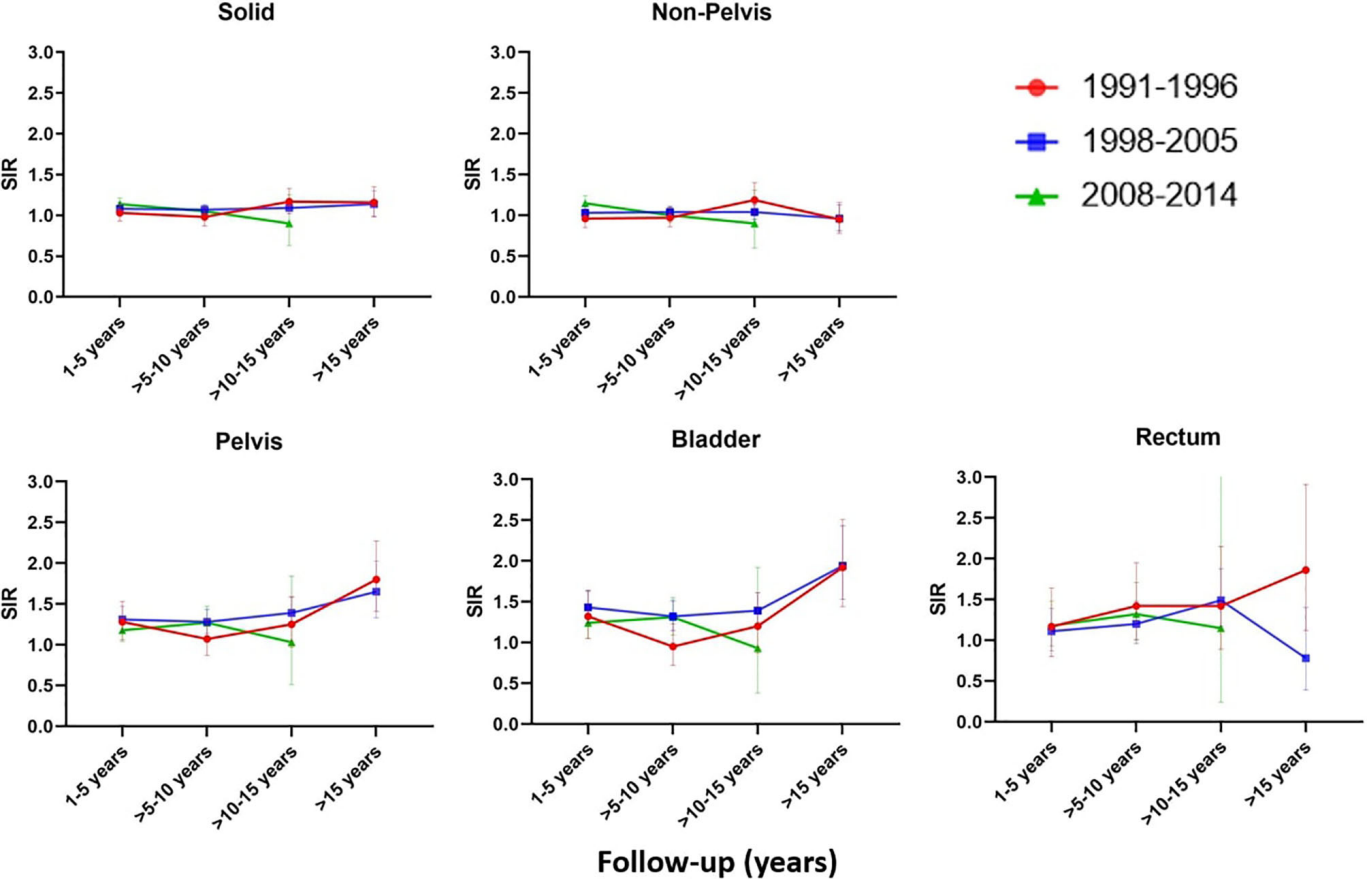
SIR for the EBRT cohort compared to the Dutch male general population for different follow-up years and time periods.

Analysis stratified by follow-up years and time period of diagnosis revealed a significant increase of non-pelvic SPC in the first five years of follow-up for the AdvRT era (SIR=1.15 (1.07-1.24), AER=19.76) (**Figure 1** and **Supplementary Table 3**). Second pelvic cancers were significantly increased for all follow-up years for the 3D-CRT era, with the biggest increase being observed >15 years of follow-up (SIR=1.65 (1.33-2.03), AER=35.39).

### Comparison of the EBRT Cohort to the Radical Prostatectomy Cohort

For the total EBRT cohort the adjusted sHR (95% confidence interval and p-value) (EBRT vs. radical prostatectomy) for developing a solid SPC was 1.24 (1.19-1.30, p=<0.01) (**Table 3**). The risk for developing a solid SPC was significantly elevated in the EBRT cohort for all time periods, compared to the radical prostatectomy cohort. The risk for developing a non-pelvic SPC was highest for the last time period 1.17 (1.06-1.29, p=<0.01). For second cancers in the pelvic region, the risk was highest in the second time period 1.74 (1.52-2.00, p=<0.01), followed by the last time period 1.47 (1.24-1.74, p=<0.01). More detailed information on comparison of the EBRT cohort with the prostatectomy cohort can be found in **Table 3**.

## Discussion

The complete PCa survivor population had a small, not statistically significant reduced risk of developing a SPC. In PCa patients treated with EBRT an 8% increased risk of developing a solid SPC was observed, which corresponds with an absolute excess number of 14.5 patients diagnosed with a second cancer per 10.000, compared to the Dutch male general population. This risk was particularly evident for SPC within the pelvic region.

The reduced risk of developing a SPC in the complete PCa survivor population is partially in line with findings from other studies (2, 10, 20, 21). Davis et al. (20), carried out a population-based cohort study in the US, and found that the risk of developing a SPC for the complete PCa patient population is significantly reduced (20). They related this reduction in risk to the younger age of patients at time of diagnosis, and the routine screening of the prostate-specific antigen (PSA). In the Netherlands, men are not actively screened on PSA. This may explain as to why the observed risk of developing a SPC was not as significantly reduced in our complete PCa survivor population. Nonetheless, PCa detected by opportunistic screening as applied in the Netherlands most likely represent men with higher socio economic status, which is generally associated with a lower cancer risk. Men of higher socio economic status might be more health conscious than the general population (21).

Several cohort studies have previously reported on the increased risk of developing a SPC in the pelvic area after EBRT for PCa (5–8, 20). Organs within the pelvis (e.g. bladder and rectum), inevitably receive radiation dose due to their close proximity to the prostate. This increases the likelihood of developing a SPC in those organs. In this study, we have shown that the risk for a pelvic SPC is highest in patients treated in the second time period, corresponding to the 3D-CRT era. We also showed that a significant increase in risk persists over the years, indicating that also after AdvRT there is a higher risk of developing a pelvic SPC. A study by Zelefsky et al. (15), which investigated SPC rates after PCa found lower incidence rates of secondary bladder and rectal cancers after treatment with IMRT (15). However, no comparison was done with a 3D-CRT cohort. In a previous single-center study, where we investigated SPC risk after IMRT vs 3D-CRT, we observed no significant differences in overall pelvis SPC risks between the 2 techniques, with a trend for IMRT of lower bladder SPC risks and higher rectum SPC risks (22).

We observed an increase in non-pelvic SPC for the most recent RT period. Although the high-dose region is more compact with AdvRT and more conformal dose distribution can be achieved (sparing nearby structures such as bladder and rectum, from intermediate- to high-dose volumes), the lower dose region is expanded due to increased beam angles, exposing more normal tissue to a low-dose bath. Therefore, AdvRT is at the expense of a larger volume of more distant tissues receiving low-to-moderate doses compared to more conformal RT (16, 17). The results of this study as well as the theoretical concerns support the findings from the previously carried out single center study in which we observed that patients treated with IMRT had a significantly increased risk for non-pelvic cancers as opposed to those treated with 3D-CRT, especially in survivors aged <70 and active smokers at time of treatment (22).

In the current study, we found a significant increase in second rectum cancers for the AdvRT time period. This finding is in agreement with the finding of our previous single-center study, but is contradictory to the findings of Journy et al., who observed a reduced risk for second rectum cancers after treatment with IMRT (17). These findings were based on sufficient follow-up to monitor early incidence of SPC risk, however, are limited by follow-up (median follow-up: 5.2 years). Our analysis by follow-up period revealed that the risk for rectum SPC only significantly increased after 5 years of follow-up. This observation was also described in other cohort studies (2, 23–25).

We furthermore found that PCa patients treated with EBRT had a 22% increased risk of developing a second hematological cancer (AER=4.39). Second hematological cancers are less well described in literature as opposed to second solid cancers. This is partially attributed to the fact that the absolute numbers of hematological cancers are relatively low in the general population. Therefore, large study populations and sufficient follow-up is required to investigate second hematological cancer risk. Studies reporting on second hematological cancer risk, report similar findings to those we made in this study; namely elevated risks after EBRT (17, 24). We are currently busy with carrying out a follow-up study, exploring hematological cancer risk after EBRT for PCa further. In this follow-up study, we will also specifically look into different subtypes of hematological cancers.

In line with observations from epidemiological studies (26–28), we found that younger age is associated with increased SPC risks. This can be explained by the biological phenomenon that cells of older people are less sensitive to radiation (26, 27). A study by de Gonzalez et al., exploring SPC risk after RT for different cancer sites, found that the relative risk for second cancers is increased with younger age at diagnosis (24). The relative risk for SPC after PCa was reported to decrease from 1.85 (95% CI = 1.53-2.22) in patients aged below 60 years to 1.16 (95% CI = 0.96-1.14) in patients aged >75 years. In the present study we found that the risk for developing a solid SPC decreased by 10% in patients aged >70 years.

The fact that PCa survivors in the three defined RT groups were treated in different calendar periods might be associated with potential confounding effects such as e.g. differences in patient populations selected for RT, differences in targeted volumes, differences in follow-up intensity/follow-up imaging, and differences in adjuvant or later treatment during follow-up (e.g. hormonal treatment, chemotherapy). In our previous single-center study, we were able to investigate several potential confounders, such as the prescription of adjuvant HT. At sensitivity analysis, adjuvant HT demonstrated to be not affecting the results of the analysis (22). Hence, for this study we included both patients - with and without adjuvant HT prescription to the EBRT cohort. We planned to obtain a similar detailed database for an extended patient group of several hospitals, to investigate this further with more statistical power.

The major strengths of this study are the large sample size, its ability to assess trends over time, and the fact that a dual comparison was drawn (Dutch general population and radical prostatectomy cohort). The reported results from the two methods were roughly in agreement, identifying similar trends in SPC risk after PCa diagnosis. The main limitation of this study is that no comprehensive RT information was available. The time periods defined act as a proxy for the different EBRT techniques used. Over the years there have been multiple changes in the field of EBRT, ranging from the dose and fractions prescribed to the use of image-guidance. In this study we were unable to take these factors into consideration. However, we are currently busy conducting a study, exploring how specific characteristics of EBRT impact the risk of developing a SPC. Furthermore, we were unable to the explore the effect of smoking on SPC risk, as this information is not recorded in the NCR. Smoking is a known risk factor for the development of cancers, such as bladder cancer. Even though it is not a known risk factor for the development of PCa, some studies have also shown that smoking and RT are interactive factors, affecting the risk of developing a SPC. Lastly, the AdvRT era is limited by its follow-up. We were unable to generate a thorough risk assessment on the effect AdvRT has on the development of a SPC beyond 10 years of follow-up (22, 29, 30).

In conclusion, PCa patients who received EBRT had a significantly increased risk of developing a SPC compared to both the general population and the radical prostatectomy cohort. The results indicate that over the years, the risk for second pelvic cancers persists and the risk for second non-pelvic cancers increases. Younger age at point of diagnosis increases the risk of developing a SPC. These results confirm what was previously described in other studies and underline the importance of providing sufficient follow-up care, especially considering the high survival prospects of PCa survivors. Further research containing more detailed RT information, as well as exploring the risk of developing a second hematological cancer after EBRT for PCa, is currently ongoing.

## Data Availability Statement

The raw data supporting the conclusions of this article will be made available by the authors, without undue reservation.

## Author Contributions

KA, WH, LI, and MH contributed to the study design. M-CJ, KA, WH, and BS contributed to data collection and analysis. All authors participated in data interpretation and revision. M-CJ, WH, and KA contributed to writing the manuscript. KA and WH contributed to supervision and study management. All authors contributed to the article and approved the submitted version.

## Funding

For this project a grant was received from the Dutch Cancer Society (KWF), nr 12009. The funder of the study is a non-profit organization and it had no role in study design, data collection, data analysis, data interpretation, or writing of the report.

## Conflict of Interest

The authors declare that the research was conducted in the absence of any commercial or financial relationships that could be construed as a potential conflict of interest.

## Publisher’s Note

All claims expressed in this article are solely those of the authors and do not necessarily represent those of their affiliated organizations, or those of the publisher, the editors and the reviewers. Any product that may be evaluated in this article, or claim that may be made by its manufacturer, is not guaranteed or endorsed by the publisher.
