# Supplementary material for: Impact of Advanced Radiotherapy on Second Primary Cancer Risk in Prostate Cancer Survivors: A Nationwide Cohort Study
Source: Front Oncol. 2021 Nov 26;11:771956. doi: 10.3389/fonc.2021.771956 (PMC8662556; doi:10.3389/fonc.2021.771956)
Supplement: Supplementary file 1 [file Table_1.docx]

**Supplementary Material: Impact of advanced radiotherapy on second primary cancer risk in prostate cancer survivors: a nationwide cohort study**

Supplementary Table 1. SIR and AER (per 10,000 person years) for the complete PCa cohort compared to the Dutch general population.

|  | Observed (n) | Expected (n) | SIR (95% CI) | AER |
| --- | --- | --- | --- | --- |
| All SPC | 22434 | 22807.7 | 0.98 (0.97-1.00) | -3.19 |
| Hematological | 2567 | 2347.9 | 1.09 (1.05-1.14)* | 1.78 |
| All solid SPC | 20179 | 20680 | 0.98 (0.96-0.99) | -4.25 |
| Pelvis | 5702 | 5289.1 | 1.08 (1.05-1.11)* | 3.40 |
| Non-Pelvis | 15199 | 15784.3 | 0.96 (0.95-0.98) | -4.88 |
| All solid SPC by anatomical region |  |  |  |  |
| Pelvis |  |  |  |  |
| Male genital organs | 111 | 108.6 | 1.02 (0.84-1.23) | 0.02 |
| Bladder | 3782 | 3512.9 | 1.08 (1.04-1.11)* | 1.95 |
| Urethra | 77 | 70.1 | 1.10 (0.87-1.37) | 0.05 |
| Rectum | 1745 | 1582.3 | 1.10 (1.05-1.15)* | 1.17 |
| Non-Pelvis |  |  |  |  |
| Neck and up | 1299 | 1457.2 | 0.89 (0.84-0.94) | -1.14 |
| Chest | 5255 | 5792.1 | 0.91 (0.88-0.93) | -3.86 |
| Lung & bronchus | 4740 | 5340.7 | 0.89 (0.86-0.91) | -4.32 |
| Male breast | 49 | 61.1 | 0.80 (0.59-1.06) | -0.09 |
| Abdomen | 7548 | 7535.2 | 1.00 (0.98-1.02) | 0.09 |
| Esophagus | 808 | 857 | 0.94 (0.88-1.01) | -0.35 |
| Stomach | 678 | 652.7 | 1.04 (0.96-1.12) | 0.18 |
| Small intestine | 33 | 28.7 | 1.15 (0.79-1.61) | 0.03 |
| Colon | 3496 | 3320 | 1.05 (1.02-1.09)* | 1.25 |
| Liver | 189 | 240.3 | 0.79 (0.68-0.91) | -0.37 |
| Pancreas | 812 | 773.1 | 1.05 (0.98-1.13) | 0.28 |
| Kidney | 757 | 775.9 | 0.98 (0.91-1.05) | -0.14 |
| Renal pelvis | 166 | 178.7 | 0.93 (0.79-1.08) | -0.09 |
| Ureter | 161 | 178.5 | 0.90 (0.77-1.05) | -0.13 |
| Other | | | | |
| Central Nervous System | 298 | 278.9 | 1.07 (0.95-1.20) | 0.14 |
| Melanoma | 1248 | 1011.5 | 1.23 (1.17-1.30)* | 1.70 |
| Bone | 31 | 20.5 | 1.51 (1.03-2.15)* | 0.08 |

*indicates significant SIRs; standarized incidence ratio (SIR); absolute excess risk (AER)

Supplementary Table 2. SIR and AER (per 10,000 person years) for the EBRT PCa cohort compared to the Dutch general population for different time periods.

|  | 1991-1996 | | | | 1998-2005 | | | | 2008-2014 | | | |
| --- | --- | --- | --- | --- | --- | --- | --- | --- | --- | --- | --- | --- |
|  | Obs (n) | Exp (n) | SIR (95%CI) | AER | Obs (n) | Exp (n) | SIR (95%CI) | AER | Obs (n) | Exp (n) | SIR (95%CI) | AER |
| All SPC | 1248 | 1163.5 | 1.07 (1.01-1.13)* | 14.74 | 3209 | 2927.7 | 1.10 (1.06-1.14)* | 20.12 | 1809 | 1630.4 | 1.11 (1.06-1.16)* | 22.38 |
| Hematological SPC | 134 | 104.9 | 1.28 (1.07-1.51)* | 4.61 | 385 | 311.1 | 1.24 (1.12-1.37)* | 4.97 | 206 | 172.8 | 1.19 (1.03-1.37)* | 3.98 |
| All solid SPC | 1129 | 1069.4 | 1.06 (0.99-1.12) | 10.19 | 2872 | 2649.1 | 1.08 (1.04-1.12)* | 15.81 | 1591 | 1452.6 | 1.10 (1.04-1.15)* | 17.23 |
| Non-Pelvis | 822 | 817.4 | 1.01 (0.94-1.08) | 0.78 | 2098 | 2037.5 | 1.03 (0.99-1.07) | 4.01 | 1206 | 1115.9 | 1.08 (1.02-1.14)* | 12.06 |
| Pelvis | 357 | 278.4 | 1.28 (1.15-1.42)* | 13.21 | 929 | 690.1 | 1.35 (1.26-1.44)* | 16.37 | 440 | 362.5 | 1.21 (1.10-1.33)* | 9.37 |
| All solid SPC by anatomical region | | | | | | | | | | | | |
| Pelvis | | | | | | | | | | | | |
| Male genital organs | 6 | 5.1 | 1.18 (0.43-2.45) | 0.15 | 13 | 13.6 | 0.96 (0.51-1.64) | -0.04 | 7 | 7.6 | 0.92 (0.37-1.90) | -0.07 |
| Bladder | 240 | 189.5 | 1.27 (1.11-1.44)* | 8.43 | 662 | 465.5 | 1.42 (1.32-1.53)* | 13.37 | 299 | 238 | 1.26 (1.12-1.41)* | 7.34 |
| Urethra | 3 | 3.5 | 0.86 (0.18-2.51) | -0.08 | 16 | 9.5 | 1.68 (0.96-2.74) | 0.43 | 3 | 5.4 | 0.56 (0.11-1.62) | -0.29 |
| Rectum | 112 | 80.7 | 1.39 (1.14-1.67)* | 5.17 | 246 | 203.4 | 1.21 (1.06-1.37)* | 2.86 | 135 | 109.3 | 1.24 (1.04-1.46)* | 3.07 |
| Non-Pelvis | | | | | | | | | | | | |
| Neck and up | 68 | 73.6 | 0.92 (0.72-1.17) | -0.92 | 187 | 181.9 | 1.03 (0.89-1.19) | 0.34 | 99 | 101.6 | 0.97 (0.79-1.18) | -0.31 |
| Chest | 364 | 382.3 | 0.95 (0.86-1.06) | -3.03 | 909 | 884.7 | 1.03 (0.96-1.10) | 1.63 | 489 | 450.9 | 1.08 (0.99-1.19) | 4.57 |
| Lung & bronchus | 307 | 325.5 | 0.94 (0.84-1.06) | -3.06 | 732 | 715.3 | 1.02 (0.95-1.10) | 1.12 | 381 | 351.5 | 1.08 (0.98-1.20) | 3.53 |
| Male breast | 2 | 2.7 | 0.74 (0.09-2.68) | -0.11 | 6 | 7.8 | 0.77 (0.28-1.67) | -0.12 | 2 | 4.5 | 0.44 (0.05-1.61) | -0.30 |
| Esophagus | 39 | 36.2 | 1.08 (0.77-1.47) | 0.46 | 108 | 109.1 | 0.99 (0.81-1.20) | -0.07 | 69 | 66.6 | 1.04 (0.81-1.31) | 0.29 |
| Abdomen | 362 | 336.4 | 1.08 (0.97-1.19) | 4.27 | 883 | 871.2 | 1.01 (0.95-1.08) | 0.80 | 554 | 484.8 | 1.14 (1.05-1.24)* | 8.37 |
| Stomach | 52 | 47.5 | 1.09 (0.82-1.44) | 0.74 | 83 | 86.5 | 0.96 (0.76-1.19) | -0.23 | 35 | 35.5 | 0.99 (0.69-1.37) | -0.06 |
| Small intestine | 4 | 1.2 | 3.33 (0.91-8.53) | 0.46 | 4 | 3.7 | 1.08 (0.29-2.77) | 0.02 | 2 | 2.3 | 0.87 (0.11-3.14) | -0.04 |
| Colon | 185 | 161.4 | 1.15 (0.99-1.32) | 3.91 | 454 | 437 | 1.04 (0.95-1.14) | 1.15 | 305 | 240.8 | 1.27 (1.13-1.42)* | 7.73 |
| Liver | 4 | 8.2 | 0.49 (0.13-1.25) | -0.69 | 29 | 28.2 | 1.03 (0.69-1.48) | 0.05 | 21 | 22.6 | 0.93 (0.58-1.42) | -0.19 |
| Pancreas | 30 | 36.6 | 0.82 (0.55-1.17) | -1.08 | 118 | 98.8 | 1.19 (0.99-1.43) | 1.28 | 63 | 59.8 | 1.05 (0.81-1.35) | 0.38 |
| Kidney | 47 | 34.6 | 1.36 (1.00-1.81) | 2.04 | 104 | 99.7 | 1.04 (0.85-1.26) | 0.29 | 65 | 59.2 | 1.10 (0.85-1.40) | 0.69 |
| Renal pelvis | 5 | 8.1 | 0.62 (0.20-1.44) | -0.51 | 15 | 23.3 | 0.64 (0.36-1.06) | -0.55 | 20 | 13.7 | 1.50 (0.89-2.26) | 0.75 |
| Ureter | 7 | 7.5 | 0.93 (0.38-1.92) | -0.08 | 27 | 24.1 | 1.12 (0.74-1.63) | 0.19 | 11 | 14.1 | 0.78 (0.39-1.40) | -0.37 |
| Other | | | | | | | | | | | | |
| Central Nervous System | 10 | 12.6 | 0.79 (0.38-1.46) | -0.43 | 33 | 35 | 0.94 (0.65-1.32) | -0.13 | 29 | 20.6 | 1.41 (0.94-2.02) | 1.00 |
| Melanoma | 39 | 29 | 1.34 (0.96-1.84) | 1.65 | 151 | 117.6 | 1.28 (1.09-1.51)* | 2.24 | 99 | 92.3 | 1.07 (0.87-1.31) | 0.80 |
| Bone | 2 | 0.8 | 2.50 (0.30-9.03) | 0.20 | 7 | 2.7 | 2.60 (1.04-5.34)* | 2.59 | 2 | 1.5 | 1.33 (0.16-4.82) | 0.06 |

*indicates significant SIRs; observed (Obs); expected (Exp); standarized incidence ratio (SIR); absolute excess risk (AER)

Supplementary Table 3. SIR and AER (per 10,000 person years) for the EBRT cohort compared to the Dutch general population for different follow-up years and time periods.

|  | Time Periods | | | | | | | | | | | |
| --- | --- | --- | --- | --- | --- | --- | --- | --- | --- | --- | --- | --- |
| Follow-up years | 1991-1996 | | | | 1998-2005 | | | | 2008-2014 | | | |
|  | Obs | Exp | SIR (95%CI) | AER | Obs | Exp | SIR (95%CI) | AER | Obs | Exp | SIR (95%CI) | AER |
| All Solid | 1129 | 1069.4 | 1.06 (0.99-1.12) | 10.19 | 2872 | 2649.1 | 1.08 (1.04-1.12)* | 15.81 | 1591 | 1452.6 | 1.10 (1.04-1.15)* | 17.23 |
| 1-5 years | 410 | 398.1 | 1.03 (0.93-1.13) | 5.08 | 918 | 846.4 | 1.08 (1.02-1.16)* | 13.72 | 945 | 829.2 | 1.14 (1.07-1.21)* | 24.43 |
| >5-10 years | 333 | 340.8 | 0.98 (0.87-1.09) | -4.23 | 1017 | 954.2 | 1.07 (1.00-1.13) | 12.86 | 611 | 584.5 | 1.05 (0.96-1.13) | 8.60 |
| >10-15 years | 227 | 193.8 | 1.17 (1.02-1.33)* | 35.11 | 718 | 655.8 | 1.09 (1.02-1.18)* | 20.25 | 35 | 38.9 | 0.90 (0.63-1.25) | -18.40 |
| >15 years | 158 | 136.6 | 1.16 (0.98-1.35) | 33.86 | 219 | 192.7 | 1.14 (0.99-1.30) | 28.52 | - | - | - | - |
| All hematological | 134 | 104.9 | 1.28 (1.07-1.51)* | 4.61 | 385 | 311.1 | 1.24 (1.12-1.37)* | 4.97 | 206 | 172.8 | 1.19 (1.03-1.37)* | 3.98 |
| 1-5 years | 30 | 29.5 | 1.02 (0.69-1.45) | 0.21 | 114 | 87.9 | 1.30 (1.07-1.56)* | 4.91 | 123 | 99 | 1.24 (1.03-1.48)* | 4.94 |
| >5-10 years | 47 | 33.2 | 1.42 (1.04-1.88)* | 7.14 | 135 | 113.9 | 1.19 (0.99-1.40) | 4.10 | 81 | 70.2 | 1.15 (0.92-1.43) | 3.31 |
| >10-15 years | 38 | 23.9 | 1.59 (1.13-2.18)* | 13.73 | 116 | 85.3 | 1.36 (1.12-1.63)* | 9.13 | 2 | 3.6 | 0.56 (0.07-2.01) | -6.96 |
| >15 years | 19 | 18.3 | 1.04 (0.63-1.62) | 0.97 | 20 | 24 | 0.83 (0.51-1.29) | -3.82 | - | - | - | - |
| Non-Pelvis | 822 | 817.4 | 1.01 (0.94-1.08) | 0.78 | 2098 | 2037.5 | 1.03 (0.99-1.07) | 4.01 | 1206 | 1115.9 | 1.08 (1.02-1.14)* | 12.06 |
| 1-5 years | 293 | 304.9 | 0.96 (0.85-1.08) | -5.04 | 661 | 641.6 | 1.03 (0.95-1.11) | 3.69 | 729 | 634.6 | 1.15 (1.07-1.24)* | 19.76 |
| >5-10 years | 252 | 258.8 | 0.97 (0.86-1.10) | -3.61 | 760 | 732.8 | 1.04 (0.96-1.11) | 5.43 | 450 | 451.4 | 1.00 (0.91-1.09) | -0.44 |
| >10-15 years | 176 | 147.9 | 1.19 (1.02-1.40)* | 28.67 | 532 | 511.8 | 1.04 (0.95-1.13) | 6.29 | 27 | 29.9 | 0.90 (0.60-1.31) | -13.21 |
| >15 years | 101 | 105.8 | 0.95 (0.78-1.16) | -7.18 | 145 | 151.3 | 0.96 (0.81-1.13) | -6.41 | - | - | - | - |
| Pelvis | 357 | 278.4 | 1.28 (1.15-1.42)* | 13.21 | 929 | 690.1 | 1.35 (1.26-1.44)* | 16.37 | 440 | 362.5 | 1.21 (1.10-1.33)* | 9.37 |
| 1-5 years | 123 | 96 | 1.28 (1.06-1.53)* | 11.38 | 278 | 212.3 | 1.31 (1.16-1.47)* | 12.43 | 239 | 202.4 | 1.18 (1.04-1.34)* | 7.57 |
| >5-10 years | 96 | 89.4 | 1.07 (0.87-1.31) | 3.48 | 316 | 247.4 | 1.28 (1.14-1.43)* | 13.57 | 190 | 149.4 | 1.27 (1.10-1.47)* | 12.63 |
| >10-15 years | 67 | 53.6 | 1.25 (0.97-1.59) | 13.46 | 245 | 175.8 | 1.39 (1.22-1.58)* | 21.25 | 11 | 10.7 | 1.03 (0.51-1.84) | 1.34 |
| >15 years | 71 | 39.4 | 1.80 (1.41-2.27)* | 46.02 | 90 | 54.6 | 1.65 (1.33-2.03)* | 35.39 | - | - | - | - |
| Bladder | 240 | 189.5 | 1.27 (1.11-1.44)* | 8.43 | 662 | 465.5 | 1.42 (1.32-1.53)* | 13.37 | 299 | 238 | 1.26 (1.12-1.41)* | 7.34 |
| 1-5 years | 85 | 64.4 | 1.32 (1.05-1.63)* | 8.67 | 197 | 137.8 | 1.43 (1.24-1.64)* | 11.18 | 161 | 130.2 | 1.24 (1.05-1.44)* | 6.35 |
| >5-10 years | 57 | 60.2 | 0.95 (0.72-1.23) | -1.68 | 220 | 166.5 | 1.32 (1.15-1.51)* | 10.51 | 131 | 100.3 | 1.31 (1.09-1.55)* | 9.48 |
| >10-15 years | 44 | 36.8 | 1.20 (0.87-1.61) | 7.15 | 169 | 122 | 1.39 (1.18-1.61)* | 14.26 | 7 | 7.5 | 0.93 (0.38-1.92) | -2.20 |
| >15 years | 54 | 28.1 | 1.92 (1.44-2.51)* | 37.13 | 76 | 39.2 | 1.94 (1.53-2.43)* | 36.28 | - | - | - | - |
| Rectum/Rectosigmoid | 112 | 80.7 | 1.39 (1.14-1.67)* | 5.17 | 246 | 203.4 | 1.21 (1.06-1.37)* | 2.86 | 135 | 109.3 | 1.24 (1.04-1.46)* | 3.07 |
| 1-5 years | 33 | 28.3 | 1.17 (0.80-1.64) | 1.97 | 74 | 66.9 | 1.11 (0.87-1.39) | 1.33 | 75 | 63.6 | 1.18 (0.93-1.48) | 2.35 |
| >5-10 years | 38 | 26.7 | 1.42 (1.01-1.95)* | 5.85 | 88 | 73.5 | 1.20 (0.96-1.48) | 2.81 | 57 | 43.1 | 1.32 (1.00-1.71) | 4.25 |
| >10-15 years | 22 | 15.5 | 1.42 (0.89-2.15) | 6.34 | 73 | 48.9 | 1.49 (1.17-1.88)* | 7.14 | 3 | 2.6 | 1.15 (0.24-3.37) | 1.74 |
| >15 years | 19 | 10.2 | 1.86 (1.12-2.91)* | 12.26 | 11 | 14.1 | 0.78 (0.39-1.40) | -2.94 | - | - | - | - |

*indicates significant SIRs; observed (Obs); expected (Exp); standarized incidence ratio (SIR); absolute excess risk (AER)
